# Augmented Reality-Assisted Pancreaticoduodenectomy with Superior Mesenteric Vein Resection and Reconstruction

**DOI:** 10.1155/2021/9621323

**Published:** 2021-03-17

**Authors:** Rui Tang, Wei Yang, Yucheng Hou, Lihan Yu, Guangdong Wu, Xuan Tong, Jun Yan, Qian Lu

**Affiliations:** Hepatopancreatobiliary Center, Beijing Tsinghua Changgung Hospital, School of Clinical Medicine, Institute for Precision Medicine, Tsinghua University, Beijing 102218, China

## Abstract

**Introduction:**

Pancreaticoduodenectomy (PD) with superior mesenteric vein (SMV) reconstruction are often required to achieve complete (R0) resection for pancreatic head cancer (PHC) with tumor invasion of the SMV. Augmented reality (AR) technology can be used to assist in determining the extent of SMV involvement by superimposing virtual 3-dimensional (3D) images of the pancreas and regional vasculature on the surgical field.

**Materials and Methods:**

Three patients with PHC and tumor invasion of the SMV underwent AR-assisted PD with SMV resection and reconstruction following preoperative computed tomography scanning. Preoperative imaging data were used to reconstruct 3D images of anatomical structures, including the tumor, portal vein (PV), SMV, and splenic vein (SV). Using AR software installed on a smart phone, the reconstructed 3D images were superimposed on the surgical field as viewed in a smart phone display to provide intermittent navigational assistance to the surgeon in identifying the boundaries of PHC tumor invasion for resection of the vessels involved.

**Result:**

All patients successfully completed the operation. Intraoperative AR applications displayed virtual images of the pancreas, SMV, bile duct, common hepatic artery (CHA), and superior mesenteric artery (SMA). Two patients required end-to-end anastomosis for reconstruction of the SMV. One patient required allogenic vascular bypass to reconstruct the SMV-PV juncture with concomitant reconstruction of the SV-SMV confluence by end-to-side anastomosis of the SV and bypass vessel. Postoperative pathology confirmed R0 resections for all patients.

**Conclusion:**

AR navigation technology based on preoperative CT image data can assist surgeons performing PD with SMV resection and reconstruction.

## 1. Introduction

Pancreaticoduodenectomy (PD) is a surgical procedure that is used for the resection of pancreatic head cancer (PHC), which often invades the superior mesenteric vein (SMV) [1]. Previous studies have shown that PD combined with SMV resection and reconstruction improves the rate of R0 resection and long-term prognosis of PHC patients with SMV involvement [2]. However, PHC with SMV involvement is often unresectable due to extensive tumor invasion, compression, or local adhesion. These conditions can confound efforts to locate the SMV and identify its bifurcations in the surrounding tissues. Determining the boundaries of PHC tumor invasion is critical to surgical outcome in such cases because excision of the SMV too near the site of tumor invasion increases the risk of recurrence, whereas SMV excision further from the tumor increases the difficulty of reconstructing the anastomosis.

Augmented reality (AR) technology can be used to generate 3-dimensional (3D) images from preoperative imaging data and superimpose the reconstructed 3D images on the surgical field during the intraoperative period [3]. The superimposition of the “transparent” images of anatomical structures on the surgical field assists the surgeon in determining the location of the corresponding unseen structures lying beneath the visible surfaces of organs and dissected tissues view by the surgeon [4]. The use of AR technology to assist in the identification of the inferior pancreaticoduodenal artery (IPDA) in the artery-first approach for PD has been previously reported [5]. Herein, we describe three case studies in which AR technology was used for intraoperative assistance in SMV resection and reconstruction in PHC patients undergoing PD.

## 2. Materials and Methods

### 2.1. Patients

Three patients with a preoperative diagnosis of PHC underwent PD combined with SMV resection and reconstruction in our hospital. Patients 1 (invasion length: 4 mm) and 3 (invasion length: 7 mm) were men, ages 53 and 69 years, respectively. Patient 2 (invasion length: 48 mm) was a 29-year-old woman. At the time of diagnosis, Patients 1 and 2 presented with obstructive jaundice, and Patient 3 presented with epigastric discomfort. Preoperative total blood bilirubin for Patients 1, 2, and 3, were 229, 450, and 36.8 *μ*mol/L, respectively. Our study was approved by the Ethics Committee of the Beijing Tsinghua Changgung Hospital (approval no. 20151109-04), and written informed consent has been obtained from all patients.

### 2.2. Preoperative Imaging

**Figure 1:**
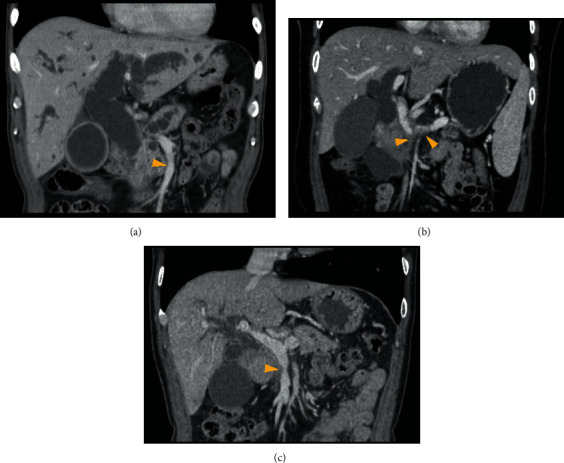
Preoperative computed tomography scans for Patients 1, 2, and 3 (a–c). (a) SMV invaded by a pancreatic tumor; (b) SMV and splenic vein invaded by pancreatic head tumor; (c) SMV invaded by pancreatic head tumor. Yellow arrowheads indicate areas of tumor invasion of blood vessels. SMV: superior mesenteric vein.

Patients underwent abdominal enhanced computed tomography (CT) scanning, in which DICOM data were obtained using a slice thickness of 1.25 mm. In each case, the CT scan revealed a space-occupying lesion in the head of the pancreas with tumor invasion of the SMV (Figure 1).

**Figure 2:**
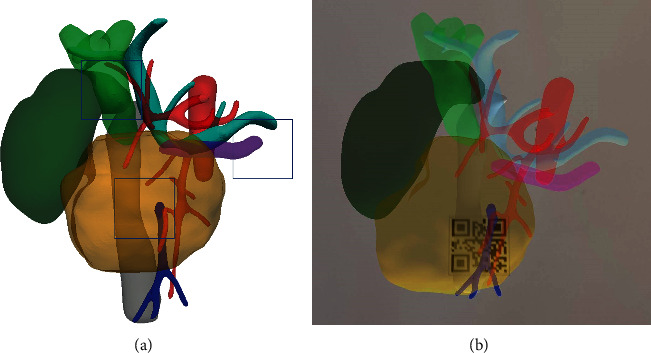
(a) 3D reconstruction and QR code setting in case of splenic vein invasion (box: QR code preset position; dark green: gallbladder; light green: common bile duct; red: artery; light blue: PV and SV; deep blue: SMV; purple: pancreatic duct). (b) The phone screen displays 3D reconstructed images and camera view after scanning the QR code in front of the tumor using AR software installed on smart phones. AR: augmented reality; PV: portal vein; QR: quick response; SMV: superior mesenteric vein; SV: splenic vein.

The Iqqa-Liver software (EDDA Technology, Princeton, USA) was used to convert the DICOM data into digital 3D image data in the STL file format, and the reconstructed 3D images were imported into the memory card of a smart phone on which the X-Liver AR software (Beijing Tsinghua Changgung Hospital, Beijing, China) had been previously installed. The X-Liver software generated a virtual 3D model of the pancreatic head and associated vasculature based on the preoperative CT image data, and a quick response (QR) code was also generated that mapped the coordinates of the 3D model. X-liver is an APP made specifically for this AR surgery. The information of the 3D model and the relationship between the model and the QR code are imported into the APP in advance, so that the corresponding 3D model will be displayed according to the location of the QR code when scanning the QR code. Three QR codes were set up in the region of the common bile duct, pancreatic head-duodenal area, and the tail of the pancreatic body. The three squares in Figure 2 are the three preset points of the QR code.

### 2.3. Registration and Image Superimposition

**Figure 3:**
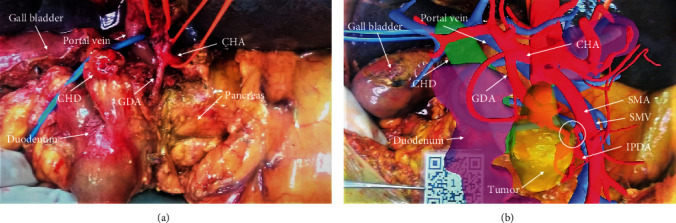
Screen capture images of the surgical field before and after QR scanning for image registration. (a) The surgical field of Patient 1 prior to the dissection of the IPDA and tumor-invaded region of the SMV. (b) Screen capture image showing placement of the QR code and superimposed virtual 3D model. The white circle indicates the region of the SMV invaded by the tumor. CHA: common hepatic artery; CHD: common hepatic duct; GDA: gastroduodenal artery; IPDA: inferior pancreaticoduodenal artery; QR: quick response; SMA: superior mesenteric artery; SMV: superior mesenteric vein.

Image registration and 3D image overlay was initiated by placing a printed copy of the QR code at the calibration position in the real surgical field, and scanning the QR code with the smart phone camera using the X-Liver software. The APP automatically recognizes the QR code and displays the images according to the preset effect. Upon scanning the QR code, the virtual 3D model was generated based on the reconstructed preoperative CT images using an artificial interactive rigid-body registration method, and the image of the 3D model was overlaid upon the image of the surgical field in smart phone display. The registration process was repeated intermittently to serve as a navigation reference for the surgeon to aid in dissection and excision placement during PD and SMV resection. After identifying the common bile duct, duodenum, and pancreas, the surgeon aligned the known structures with the 3D model so that the location of other structures in the 3D model could be used to predict and determine the approximate location of the unknown structures in the actual operation. (Figure 3).

### 2.4. Surgical Procedure

The patient took a supine position with an inverted “L” incision in the right upper quadrant. After exploring the abdominal cavity and pelvis, confirming that there are no metastases in the peritoneum, mesentery root, and liver. With the AR software the QR code was scanned to show the common hepatic arteries, the proper hepatic arteries, and the celiac axis. After a Kocher incision was used to free the duodenum and the back of the pancreatic head, the Treitz ligament was cut to reach the superior mesenteric vein and the duodenal-colonic ligament was severed to reveal the right margin of the SMV, after which the omental sac was opened. With the AR software, the QR code was scanned then to display the position of the superior mesenteric vein, splenic vein, and portal vein. Endo-GIA staplers were used to cut off the duodenal jejunum flexure and the stomach at the middle right 1/3. With the AR software, the QR code was scanned then to determine the IPDA location and the back of the pancreas was separated from the tumor-free area on the left side of the tumor, and the splenic vein was separated from the pancreas. The tail of the pancreas was sutured with a needle at the upper and lower edges of the pancreas, respectively, to facilitate hemostasis of the section when the pancreas was severed. With the AR software, the QR code was scanned and combined with intraoperative ultrasound and palpation the tumor boundary was determined. The pancreatic parenchyma was transected on the left side of the superior mesenteric vein more than 1 cm from the tumor margin. According to AR navigation, the SMV-affected area was identified; the PV, SMV, and splenic veins were blocked; and the vascular segment involved by the tumor was removed to complete the tumor en bloc resection.

**Figure 4:**
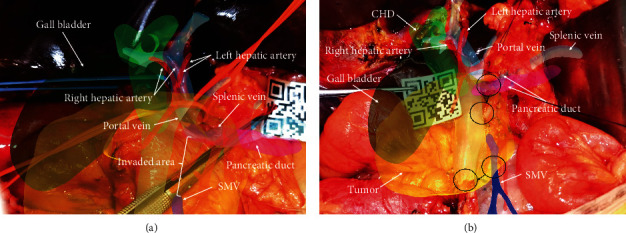
Screen capture images of surgical field of Patient 2 during resection and reconstruction of the SMV and bifurcation of the SMV and splenic vein. (a) Screen capture image showing placement of the QR code and superimposed images showing the tumor-invaded regions of the SMV and SV. (b) Screen capture image after SMV resection. The SMV was reconstructed using an allogenic donor vessel for vascular bypass of the SMV to the portal vein. An end-to-side anastomosis was used to join the splenic vein and the bypass vessel. Black circles with arrows show deviation between the locations of the blood vessels in the 3D virtual image and those of the actual structures in the surgical field. CHD: common hepatic duct; QR: quick response; SMV: superior mesenteric vein; SV: splenic vein.

**Figure 5:**
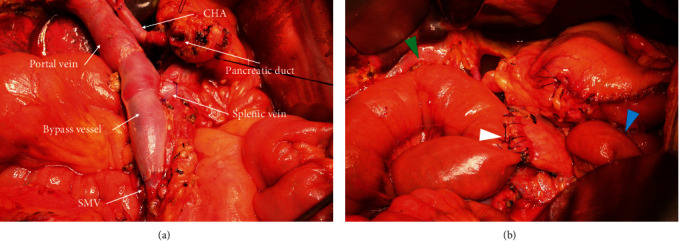
Screen capture images of the surgical field of Patient 2 following revascularization in pancreaticoduodenectomy. (a) Image showing end-to-end vascular bypass of portal vein and superior mesenteric vein (SMV) with end-to-side anastomosis of the bypass vessel and SV. (b) Image showing biliary anastomosis (green arrowhead), pancreaticojejunal anastomosis (white arrowhead), and gastrointestinal anastomosis (blue arrowhead). CHA: common hepatic artery; SMV: superior mesenteric vein; SV: splenic vein.

For Patients 1 and 3, both sides of the involved SMV segment were blocked and an end-to-end anastomosis was performed with 6-0 Prolene suture after the segment has been resected. For Patient 2, the confluence region of SV and SMV was involved and the SMV had the longest length of invasion for which an end-to-end anastomosis could not be directly performed. Therefore, PV SMV and SV were blocked, respectively, and an allogenic vascular bypass was performed, in which the two ends of an iliac vessel allograft were end-to-end anastomosed with the PV and the SMV (6-0 Prolene suture). The bypass was then laterally blocked, and an end-to-side anastomosis with the SV was performed (6-0 Prolene suture) (Figures 4 and 5).

Screen capture images of virtual 3D images superimposed on the surgical field were analyzed postoperatively to estimate deviation in the extent of SMV involvement for the AR-generated 3D model. The resection margins of the pancreas and common hepatic duct were subjected to intraoperative frozen-section pathology to confirm R0 resection.

## 3. Results

Preoperative CT showed that the length of SMV invaded by the tumor was 4, 48, and 7 mm in Patients 1, 2, and 3, respectively. In addition to the four instances indicated above at which image registration and overlay were performed, surgeons required two additional QR scans (six in total) during the course of surgery for Patients 2 and 3, which contributed to total registration times of 20, 40, and 30 min and total operation times of 9.5, 14, and 12 h for Patients 1, 2, and 3, respectively. In all 3 cases, AR software was successfully used to assist navigation for determining SMV tumor invasion area and resection and reconstruction. The estimated deviation between the boundaries of tumor invasion in the resected tissues and those represented in the virtual 3D model were 2 − 6 mm, 3 − 8 mm, and 2 − 4 mm for Patients 1, 2, and 3, respectively. Intraoperative bleeding volume was 300, 800, and 600 mL, and postoperative hospital stay was 13, 16, and 14 days for Patients 1, 2, and 3, respectively. No pancreatic fistula, postoperative hemorrhage, bile leakage, or Clavien-Dindo III-IV complications occurred. Intraoperative and postoperative pathology confirmed R0 resection margins and a diagnosis of pancreatic ductal adenocarcinoma for all three patients.

## 4. Discussion

By using AR, surgeons can predict the location of key blood vessels and tumors from known anatomical structures, making it easier to handle these structures carefully without causing accidental injury in unknown circumstances. Our AR method used preoperative CT image data to generate a transparent virtual 3D anatomical model and superimposed it on the surgical field using optical tracking and digital image registration as viewed intermittently on a smart phone display during the intraoperative period [6]. With the aid of AR navigation, the surgeon can predict the location of important blood vessels and the boundaries of organs or tumors before performing tissue dissection and organ cutting. As an emerging technology, AR has thus far been primarily applied in the field of hepatopancreatobiliary surgery, with case studies of AR-assisted hepatectomy and pancreatic surgery described in recent reports [7, 8]. This technology can also provide a visual aid to surgeons for estimating the extent of tumor invasion, establishing R0 resection margins, and avoiding secondary damage to healthy blood vessels and organs.

Pancreatic surgery often involves important blood vessels, such as the CHA, celiac trunk, superior mesenteric artery, and SMV [9]. In our current study, we used AR software to superimpose virtual 3D images of the PHC tumor, pancreas, and surrounding tissues, blood vessels, and neighboring organs on the surgical field as viewed in smart phone display to assist in surgical navigation during PD with SMV resection and reconstruction. Our results show that the use of intraoperative QR scanning for virtual 3D image registration was beneficial for aiding surgeons in determining the locations of anatomical structures that were not visible in the real surgical field at various points during SMV resection for PHC invasion.

For most PHC cases, PD is recommended for curative treatment, with R0 resection margins serving as a key predictor for optimal surgical outcome. Studies have shown that PD combined with SMV resection and reconstruction can improve the R0 resection rate of PHC and improve long-term prognosis to an extent that approaches that of patients without SMV involvement [2]. Depending on the extent of tumor invasion, resection and reconstruction of the SMV can be accomplished by resection of the vascular side-wall with local suturing or by resection of the entire blood vessel with end-to-end anastomosis. In the latter case, successful bypass may require the use of an artificial, autologous, or allogenic vessel if the length of the unresected SMV is insufficient to complete reconstruction. The 3D model generated by the AR software used in our current study provided a visual representation of the relative length of the SMV invaded by the tumor, which aided the surgeon in determining the optimal site for SMV excision for resection. Revascularization was successfully performed after resection of the lesions, including reconstruction of the SV-SMV anastomosis in Patient 2, which demonstrated that AR navigation is helpful in performing PD combined with SMV resection and reconstruction. A previous report has also suggested that AR-guided PD was beneficial for determining optimal resection margins and navigating the anatomy of the superior mesenteric artery for IPDA excision [5].

A limitation of the manual interactive registration and rigid-body registration method used in our current study is that the time required for QR code placement and image registration increase the overall intraoperative period and the deformation and displacement of organs and soft tissues leads to deviations between the transparent virtual image and the actual structure. Since in most surgeries anatomical structures are changing, the operation field is ideally constantly monitored thereby generating real-time changes to the 3D model, which is updated by perioperative CTs [10] or ultrasound images [11]. However, beside the enhanced exposure to radiation, images used in AR technology are created by complex algorithms requiring special software, which is time-consuming and novel software developments for shortening the segmentation time for overlaid image navigation are in urgent need [8]. Another approach to prevent the impact of deformation and displacement of organs is to choose anatomical landmarks, which are not highly affected by repositioning after laparotomy, dissection, or mobilization including bifurcations between major blood vessels and its branches as fiducial markers thereby improving registration errors to 6 mm [12], which is the same range of 2-8 mm achieved in our study using pancreas boundary and surrounding blood vessels as natural landmarks (Figure 4).

AR-assisted surgery will certainly become a common clinical tool for improving surgical outcomes especially in hepatopancreatobiliary surgery, though it is still in the developmental stage without fully automatic systems and standardized approaches and many clinicians are unaware of the advances in AR technology.

In conclusions, our AR navigation method provides beneficial assistance for the resection of the SMV during PD for PHC with SMV involvement. The superimposition of virtual images on the surgical field can help the surgeon determine the boundaries of tumor invasion of the SMV and neighboring vessels, thereby ensuring the success of vascular resection and reconstruction. Our results warrant further study in a larger case series to determine whether AR-assisted PD can improve the long-term prognosis of PHC patients and reduce postoperative complications, compared with conventional PD surgery.

## Data Availability

The datasets used to support the findings of this study are available from the corresponding author upon request.
